# 4-Fluoro-*N*-methyl-*N*-(1,2,3,4-tetra­hydro­carbazol-3-yl)benzene­sulfonamide

**DOI:** 10.1107/S160053680900840X

**Published:** 2009-03-11

**Authors:** Kaspar Gothardt Rasmussen, Trond Ulven, Andrew D. Bond

**Affiliations:** a University of Southern Denmark, Department of Physics and Chemistry, Campusvej 55, 5230 Odense M, Denmark

## Abstract

In the title compound, C_19_H_19_FN_2_O_2_S, the hydrogenated six-membered ring of the carbazole unit adopts a half-chair conformation and the plane of the fluoro­phenyl ring forms a dihedral angle of 41.5 (1)° with respect to the carbazole mean plane. The crystal structure is segregated into layers containing the carbazole units and fluoro­phenyl rings in alternate (200) planes. The carbazole units form centrosymmetric face-to-face inter­actions [inter­planar separation = 4.06 (1) Å] and edge-to-face inter­actions in which the N—H group is directed towards an adjacent carbazole face, with a shortest H⋯C contact of 2.53 Å. The fluoro­phenyl rings form face-to-face contacts with an approximate inter­planar separation of 3.75 Å and a centroid–centroid distance of 4.73 (1) Å.

## Related literature

For background literature and synthesis details, see: Ulven & Kostenis (2005 ▶, 2006 ▶). For related structures, see: Bjerrum *et al.* (2009 ▶); Löffler *et al.* (2009 ▶).
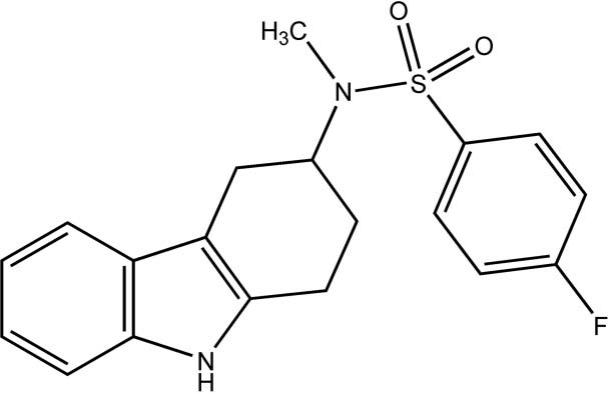


## Experimental

### 

#### Crystal data

C_19_H_19_FN_2_O_2_S
*M* *_r_* = 358.42
Monoclinic, 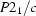

*a* = 15.2748 (7) Å
*b* = 12.0319 (6) Å
*c* = 9.4430 (4) Å
β = 102.445 (2)°
*V* = 1694.70 (14) Å^3^
*Z* = 4
Mo *K*α radiation
μ = 0.22 mm^−1^
*T* = 180 K
0.20 × 0.20 × 0.08 mm

#### Data collection

Bruker–Nonius X8 APEX-II CCD diffractometer
Absorption correction: multi-scan (*SADABS*; Sheldrick, 2003 ▶) *T*_min_ = 0.870, *T*_max_ = 0.983
29053 measured reflections
4157 independent reflections
2816 reflections with *I* > 2σ(*I*)
*R*_int_ = 0.039

#### Refinement

*R*[*F* ^2^ > 2σ(*F* ^2^)] = 0.039
*wR*(*F* ^2^) = 0.100
*S* = 1.03
4157 reflections
227 parameters
H-atom parameters constrained
Δρ_max_ = 0.28 e Å^−3^
Δρ_min_ = −0.36 e Å^−3^

### 

Data collection: *APEX2* (Bruker, 2004 ▶); cell refinement: *SAINT* (Bruker, 2003 ▶); data reduction: *SAINT*; program(s) used to solve structure: *SHELXS97* (Sheldrick, 2008 ▶); program(s) used to refine structure: *SHELXL97* (Sheldrick, 2008 ▶); molecular graphics: *SHELXTL* (Sheldrick, 2008 ▶); software used to prepare material for publication: *SHELXTL*.

## Supplementary Material

Crystal structure: contains datablocks global, I. DOI: 10.1107/S160053680900840X/gk2196sup1.cif

Structure factors: contains datablocks I. DOI: 10.1107/S160053680900840X/gk2196Isup2.hkl

Additional supplementary materials:  crystallographic information; 3D view; checkCIF report

## Figures and Tables

**Fig. 1.**
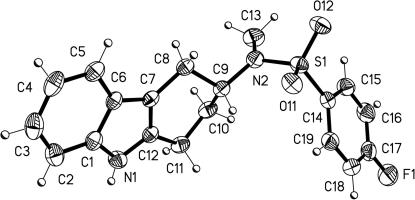
Molecular structure of the title compound with displacement ellipsoids shown at 50% probability for non-H atoms.

**Fig. 2.**
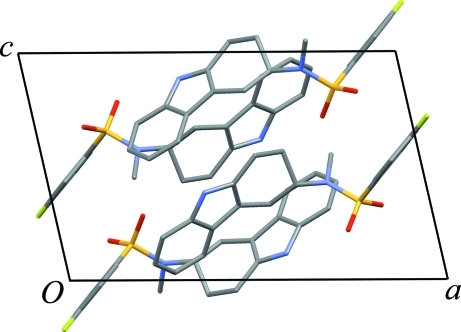
Unit-cell contents projected along the b axis, showing segregation of carbazole and fluorophenyl groups in the (200) planes. H atoms are omitted.

## supplementary crystallographic information

### Comment

The title compound is useful as an intermediate in the synthesis of antagonists of the prostaglandin D_2_ receptor CRTH2 (DP_2_) (Ulven & Kostenis, 2006).

### Experimental

The compound was synthesized as described in Ulven & Kostenis (2005).

### Refinement

H atoms bound to C atoms were placed in idealized positions with C—H = 0.95–1.00 Å and refined as riding with *U*_iso_(H) = 1.2 or 1.5*U*_eq_(C). The methyl group was allowed to rotate about its local threefold axis. The H atom of the NH group was visible in a difference Fourier map but was placed geometrically and refined as riding for the final cycles of refinement with N—H = 0.88 Å and *U*_iso_(H) = 1.2*U*_eq_(N).

### Crystal data

**Table:**

C_19_H_19_FN_2_O_2_S	*F*(000) = 752
*M**_r_* = 358.42	*D*_x_ = 1.405 Mg m^−^^3^
Monoclinic, *P*2_1_/*c*	Mo *K*α radiation, λ = 0.71073 Å
Hall symbol: -P 2ybc	Cell parameters from 6906 reflections
*a* = 15.2748 (7) Å	θ = 2.8–24.2°
*b* = 12.0319 (6) Å	µ = 0.22 mm^−^^1^
*c* = 9.4430 (4) Å	*T* = 180 K
β = 102.445 (2)°	Plate, yellow
*V* = 1694.70 (14) Å^3^	0.20 × 0.20 × 0.08 mm
*Z* = 4	

### Data collection

**Table:**

Bruker–Nonius X8 APEX-II CCD diffractometer	4157 independent reflections
Radiation source: fine-focus sealed tube	2816 reflections with *I* > 2σ(*I*)
graphite	*R*_int_ = 0.039
Thin–slice ω and φ scans	θ_max_ = 28.4°, θ_min_ = 3.6°
Absorption correction: multi-scan (*SADABS*; Sheldrick, 2003)	*h* = −20→20
*T*_min_ = 0.870, *T*_max_ = 0.983	*k* = −14→16
29053 measured reflections	*l* = −12→12

### Refinement

**Table:**

Refinement on *F*^2^	Primary atom site location: structure-invariant direct methods
Least-squares matrix: full	Secondary atom site location: difference Fourier map
*R*[*F*^2^ > 2σ(*F*^2^)] = 0.039	Hydrogen site location: inferred from neighbouring sites
*wR*(*F*^2^) = 0.100	H-atom parameters constrained
*S* = 1.03	*w* = 1/[σ^2^(*F*_o_^2^) + (0.0499*P*)^2^ + 0.197*P*] where *P* = (*F*_o_^2^ + 2*F*_c_^2^)/3
4157 reflections	(Δ/σ)_max_ = 0.001
227 parameters	Δρ_max_ = 0.28 e Å^−^^3^
0 restraints	Δρ_min_ = −0.36 e Å^−^^3^

### Special details

**Table:**

Geometry. All e.s.d.'s (except the e.s.d. in the dihedral angle between two l.s. planes) are estimated using the full covariance matrix. The cell e.s.d.'s are taken into account individually in the estimation of e.s.d.'s in distances, angles and torsion angles; correlations between e.s.d.'s in cell parameters are only used when they are defined by crystal symmetry. An approximate (isotropic) treatment of cell e.s.d.'s is used for estimating e.s.d.'s involving l.s. planes.
Refinement. Refinement of *F*^2^ against ALL reflections. The weighted *R*-factor *wR* and goodness of fit *S* are based on *F*^2^, conventional *R*-factors *R* are based on *F*, with *F* set to zero for negative *F*^2^. The threshold expression of *F*^2^ > σ(*F*^2^) is used only for calculating *R*-factors(gt) *etc*. and is not relevant to the choice of reflections for refinement. *R*-factors based on *F*^2^ are statistically about twice as large as those based on *F*, and *R*- factors based on ALL data will be even larger.

### Fractional atomic coordinates and isotropic or equivalent isotropic displacement parameters (Å^2^)

**Table:**

	*x*	*y*	*z*	*U*_iso_*/*U*_eq_	
S1	0.18551 (2)	0.58771 (3)	0.65744 (4)	0.03103 (13)	
F1	−0.04364 (7)	0.90794 (10)	0.28602 (13)	0.0644 (4)	
O11	0.23235 (7)	0.64670 (9)	0.78251 (11)	0.0366 (3)	
O12	0.13016 (7)	0.49488 (9)	0.67472 (12)	0.0418 (3)	
N1	0.58988 (8)	0.75139 (11)	0.61606 (14)	0.0345 (3)	
H1	0.6060	0.8092	0.5706	0.041*	
N2	0.26020 (8)	0.54273 (11)	0.57403 (14)	0.0332 (3)	
C1	0.64139 (10)	0.70104 (13)	0.73606 (16)	0.0310 (4)	
C2	0.72812 (10)	0.72533 (15)	0.81236 (18)	0.0392 (4)	
H2A	0.7609	0.7863	0.7862	0.047*	
C3	0.76405 (11)	0.65708 (16)	0.92717 (19)	0.0440 (5)	
H3A	0.8230	0.6713	0.9813	0.053*	
C4	0.71637 (11)	0.56773 (15)	0.96612 (18)	0.0417 (4)	
H4A	0.7435	0.5220	1.0455	0.050*	
C5	0.63012 (10)	0.54441 (14)	0.89130 (17)	0.0346 (4)	
H5A	0.5979	0.4836	0.9191	0.041*	
C6	0.59115 (9)	0.61180 (12)	0.77416 (16)	0.0275 (3)	
C7	0.50635 (10)	0.61190 (12)	0.67235 (15)	0.0260 (3)	
C8	0.42395 (9)	0.54333 (13)	0.66823 (16)	0.0295 (3)	
H8A	0.4162	0.5289	0.7680	0.035*	
H8B	0.4294	0.4711	0.6208	0.035*	
C9	0.34359 (10)	0.60775 (13)	0.58295 (16)	0.0285 (4)	
H9A	0.3378	0.6767	0.6393	0.034*	
C10	0.35930 (11)	0.64426 (13)	0.43638 (17)	0.0348 (4)	
H10A	0.3036	0.6771	0.3784	0.042*	
H10B	0.3749	0.5788	0.3833	0.042*	
C11	0.43474 (10)	0.72944 (13)	0.45470 (17)	0.0351 (4)	
H11A	0.4576	0.7338	0.3644	0.042*	
H11B	0.4116	0.8037	0.4734	0.042*	
C12	0.50906 (10)	0.69691 (13)	0.57835 (16)	0.0283 (3)	
C13	0.23280 (12)	0.46523 (15)	0.45307 (19)	0.0449 (5)	
H13A	0.2834	0.4171	0.4458	0.067*	
H13B	0.2138	0.5071	0.3627	0.067*	
H13C	0.1829	0.4195	0.4698	0.067*	
C14	0.11647 (9)	0.68484 (13)	0.54524 (16)	0.0297 (4)	
C15	0.04708 (10)	0.64712 (15)	0.43564 (18)	0.0390 (4)	
H15A	0.0367	0.5697	0.4215	0.047*	
C16	−0.00662 (11)	0.72264 (16)	0.3475 (2)	0.0452 (5)	
H16A	−0.0540	0.6983	0.2713	0.054*	
C17	0.00987 (11)	0.83361 (16)	0.3721 (2)	0.0440 (5)	
C18	0.07783 (11)	0.87337 (15)	0.4788 (2)	0.0439 (4)	
H18A	0.0874	0.9510	0.4924	0.053*	
C19	0.13208 (11)	0.79758 (14)	0.56618 (18)	0.0366 (4)	
H19A	0.1801	0.8228	0.6407	0.044*	

### Atomic displacement parameters (Å^2^)

**Table:**

	*U*^11^	*U*^22^	*U*^33^	*U*^12^	*U*^13^	*U*^23^
S1	0.0310 (2)	0.0315 (2)	0.0284 (2)	−0.00616 (17)	0.00148 (16)	−0.00040 (17)
F1	0.0426 (6)	0.0621 (8)	0.0852 (9)	0.0135 (5)	0.0064 (6)	0.0321 (6)
O11	0.0376 (6)	0.0446 (7)	0.0257 (6)	−0.0085 (5)	0.0024 (5)	−0.0055 (5)
O12	0.0394 (6)	0.0390 (7)	0.0435 (7)	−0.0114 (5)	0.0015 (5)	0.0075 (6)
N1	0.0375 (7)	0.0345 (8)	0.0320 (8)	−0.0019 (6)	0.0089 (6)	0.0072 (6)
N2	0.0310 (7)	0.0302 (7)	0.0353 (8)	−0.0023 (6)	0.0001 (6)	−0.0086 (6)
C1	0.0323 (8)	0.0345 (9)	0.0270 (8)	0.0057 (7)	0.0085 (7)	−0.0026 (7)
C2	0.0334 (9)	0.0494 (11)	0.0352 (10)	−0.0026 (8)	0.0083 (8)	−0.0054 (8)
C3	0.0327 (9)	0.0608 (13)	0.0362 (10)	0.0064 (9)	0.0025 (8)	−0.0101 (9)
C4	0.0408 (10)	0.0514 (12)	0.0292 (9)	0.0172 (9)	−0.0005 (8)	0.0005 (8)
C5	0.0403 (9)	0.0351 (9)	0.0277 (9)	0.0089 (7)	0.0059 (7)	0.0025 (7)
C6	0.0296 (8)	0.0301 (9)	0.0237 (8)	0.0066 (7)	0.0076 (6)	−0.0019 (7)
C7	0.0313 (8)	0.0260 (8)	0.0213 (8)	0.0058 (6)	0.0068 (6)	0.0001 (6)
C8	0.0337 (8)	0.0290 (8)	0.0250 (8)	0.0039 (7)	0.0044 (6)	0.0030 (6)
C9	0.0296 (8)	0.0272 (9)	0.0264 (8)	0.0005 (6)	0.0006 (6)	−0.0037 (6)
C10	0.0420 (9)	0.0326 (9)	0.0262 (8)	0.0060 (8)	−0.0007 (7)	0.0030 (7)
C11	0.0411 (9)	0.0330 (9)	0.0288 (9)	0.0049 (7)	0.0025 (7)	0.0085 (7)
C12	0.0315 (8)	0.0288 (8)	0.0249 (8)	0.0052 (7)	0.0070 (6)	0.0000 (6)
C13	0.0530 (11)	0.0365 (10)	0.0424 (11)	−0.0068 (8)	0.0040 (9)	−0.0142 (8)
C14	0.0265 (8)	0.0338 (9)	0.0296 (8)	−0.0027 (7)	0.0079 (7)	−0.0009 (7)
C15	0.0331 (9)	0.0374 (10)	0.0433 (10)	−0.0041 (8)	0.0016 (8)	−0.0004 (8)
C16	0.0312 (9)	0.0528 (12)	0.0470 (11)	−0.0038 (8)	−0.0014 (8)	0.0048 (9)
C17	0.0301 (9)	0.0495 (12)	0.0541 (12)	0.0086 (8)	0.0129 (8)	0.0181 (9)
C18	0.0402 (10)	0.0322 (10)	0.0618 (12)	0.0023 (8)	0.0167 (9)	0.0035 (9)
C19	0.0325 (8)	0.0363 (10)	0.0407 (10)	−0.0032 (7)	0.0070 (7)	−0.0035 (8)

### Geometric parameters (Å, °)

**Table:**

S1—O11	1.4305 (11)	C8—H8A	0.990
S1—O12	1.4312 (11)	C8—H8B	0.990
S1—N2	1.6131 (13)	C9—C10	1.520 (2)
S1—C14	1.7660 (16)	C9—H9A	1.000
F1—C17	1.3572 (19)	C10—C11	1.524 (2)
N1—C1	1.3733 (19)	C10—H10A	0.990
N1—C12	1.3751 (19)	C10—H10B	0.990
N1—H1	0.880	C11—C12	1.495 (2)
N2—C13	1.4640 (19)	C11—H11A	0.990
N2—C9	1.4817 (19)	C11—H11B	0.990
C1—C2	1.396 (2)	C13—H13A	0.980
C1—C6	1.411 (2)	C13—H13B	0.980
C2—C3	1.376 (2)	C13—H13C	0.980
C2—H2A	0.950	C14—C19	1.384 (2)
C3—C4	1.392 (3)	C14—C15	1.388 (2)
C3—H3A	0.950	C15—C16	1.378 (2)
C4—C5	1.383 (2)	C15—H15A	0.950
C4—H4A	0.950	C16—C17	1.369 (3)
C5—C6	1.397 (2)	C16—H16A	0.950
C5—H5A	0.950	C17—C18	1.368 (2)
C6—C7	1.437 (2)	C18—C19	1.380 (2)
C7—C12	1.361 (2)	C18—H18A	0.950
C7—C8	1.498 (2)	C19—H19A	0.950
C8—C9	1.526 (2)		
			
O11—S1—O12	119.71 (7)	N2—C9—H9A	107.1
O11—S1—N2	106.80 (6)	C10—C9—H9A	107.1
O12—S1—N2	106.93 (7)	C8—C9—H9A	107.1
O11—S1—C14	107.14 (7)	C9—C10—C11	110.79 (13)
O12—S1—C14	107.08 (7)	C9—C10—H10A	109.5
N2—S1—C14	108.84 (7)	C11—C10—H10A	109.5
C1—N1—C12	109.04 (13)	C9—C10—H10B	109.5
C1—N1—H1	125.5	C11—C10—H10B	109.5
C12—N1—H1	125.5	H10A—C10—H10B	108.1
C13—N2—C9	118.58 (13)	C12—C11—C10	109.94 (13)
C13—N2—S1	118.77 (11)	C12—C11—H11A	109.7
C9—N2—S1	119.01 (10)	C10—C11—H11A	109.7
N1—C1—C2	130.16 (15)	C12—C11—H11B	109.7
N1—C1—C6	107.44 (13)	C10—C11—H11B	109.7
C2—C1—C6	122.39 (14)	H11A—C11—H11B	108.2
C3—C2—C1	117.05 (16)	C7—C12—N1	109.95 (13)
C3—C2—H2A	121.5	C7—C12—C11	125.57 (14)
C1—C2—H2A	121.5	N1—C12—C11	124.45 (13)
C2—C3—C4	121.77 (16)	N2—C13—H13A	109.5
C2—C3—H3A	119.1	N2—C13—H13B	109.5
C4—C3—H3A	119.1	H13A—C13—H13B	109.5
C5—C4—C3	121.13 (16)	N2—C13—H13C	109.5
C5—C4—H4A	119.4	H13A—C13—H13C	109.5
C3—C4—H4A	119.4	H13B—C13—H13C	109.5
C4—C5—C6	118.89 (16)	C19—C14—C15	120.47 (15)
C4—C5—H5A	120.6	C19—C14—S1	120.04 (12)
C6—C5—H5A	120.6	C15—C14—S1	119.49 (12)
C5—C6—C1	118.77 (14)	C16—C15—C14	119.64 (16)
C5—C6—C7	134.35 (15)	C16—C15—H15A	120.2
C1—C6—C7	106.87 (13)	C14—C15—H15A	120.2
C12—C7—C6	106.70 (13)	C17—C16—C15	118.47 (16)
C12—C7—C8	122.71 (13)	C17—C16—H16A	120.8
C6—C7—C8	130.38 (13)	C15—C16—H16A	120.8
C7—C8—C9	108.04 (12)	F1—C17—C18	118.30 (17)
C7—C8—H8A	110.1	F1—C17—C16	118.42 (17)
C9—C8—H8A	110.1	C18—C17—C16	123.28 (17)
C7—C8—H8B	110.1	C17—C18—C19	118.16 (16)
C9—C8—H8B	110.1	C17—C18—H18A	120.9
H8A—C8—H8B	108.4	C19—C18—H18A	120.9
N2—C9—C10	113.79 (12)	C18—C19—C14	119.97 (16)
N2—C9—C8	110.22 (12)	C18—C19—H19A	120.0
C10—C9—C8	111.13 (12)	C14—C19—H19A	120.0
			
O11—S1—N2—C13	171.11 (11)	C7—C8—C9—N2	179.29 (12)
O12—S1—N2—C13	41.83 (14)	C7—C8—C9—C10	52.20 (16)
C14—S1—N2—C13	−73.52 (13)	N2—C9—C10—C11	168.46 (12)
O11—S1—N2—C9	−30.69 (13)	C8—C9—C10—C11	−66.42 (17)
O12—S1—N2—C9	−159.97 (11)	C9—C10—C11—C12	40.74 (18)
C14—S1—N2—C9	84.67 (12)	C6—C7—C12—N1	−0.90 (17)
C12—N1—C1—C2	179.04 (16)	C8—C7—C12—N1	174.28 (13)
C12—N1—C1—C6	0.25 (16)	C6—C7—C12—C11	−179.17 (14)
N1—C1—C2—C3	−177.84 (15)	C8—C7—C12—C11	−4.0 (2)
C6—C1—C2—C3	0.8 (2)	C1—N1—C12—C7	0.42 (17)
C1—C2—C3—C4	−0.1 (2)	C1—N1—C12—C11	178.71 (14)
C2—C3—C4—C5	−0.6 (3)	C10—C11—C12—C7	−7.3 (2)
C3—C4—C5—C6	0.5 (2)	C10—C11—C12—N1	174.63 (14)
C4—C5—C6—C1	0.2 (2)	O11—S1—C14—C19	16.81 (14)
C4—C5—C6—C7	178.64 (16)	O12—S1—C14—C19	146.41 (13)
N1—C1—C6—C5	178.01 (13)	N2—S1—C14—C19	−98.34 (14)
C2—C1—C6—C5	−0.9 (2)	O11—S1—C14—C15	−163.65 (12)
N1—C1—C6—C7	−0.79 (16)	O12—S1—C14—C15	−34.05 (14)
C2—C1—C6—C7	−179.70 (14)	N2—S1—C14—C15	81.21 (13)
C5—C6—C7—C12	−177.49 (16)	C19—C14—C15—C16	−0.1 (2)
C1—C6—C7—C12	1.04 (16)	S1—C14—C15—C16	−179.68 (13)
C5—C6—C7—C8	7.8 (3)	C14—C15—C16—C17	−0.6 (2)
C1—C6—C7—C8	−173.64 (14)	C15—C16—C17—F1	−179.37 (15)
C12—C7—C8—C9	−18.43 (19)	C15—C16—C17—C18	0.9 (3)
C6—C7—C8—C9	155.51 (15)	F1—C17—C18—C19	179.96 (14)
C13—N2—C9—C10	36.64 (19)	C16—C17—C18—C19	−0.3 (3)
S1—N2—C9—C10	−121.59 (13)	C17—C18—C19—C14	−0.5 (2)
C13—N2—C9—C8	−88.96 (16)	C15—C14—C19—C18	0.7 (2)
S1—N2—C9—C8	112.81 (12)	S1—C14—C19—C18	−179.72 (12)
